# Potential Sources of Novel Foods to Procure Nutrients and Bioactive Compounds for Disease Prevention

**DOI:** 10.3390/nu16234130

**Published:** 2024-11-29

**Authors:** María del Mar Contreras, Francisca I. Bravo, Wilman Carrillo

**Affiliations:** 1 Department of Chemical, Environmental and Materials Engineering, University of Jaén, Campus Las Lagunillas, 23071 Jaén, Spain; 2 Institute of Biorefineries Research (I3B), University of Jaén, Campus Las Lagunillas, 23071 Jaén, Spain; 3 Nutrigenomics Research Group, Department of Biochemistry and Biotechnology, Universitat Rovira i Virgili, 43007 Tarragona, Spain; franciscaisabel.bravo@urv.cat; 4 Research Department, State University of Bolívar, Guaranda 020102, Ecuador; wcarrillo@utb.edu.ec; 5 Rural and Food Engineering Department, Polytechnic University of Valencia, 46022 Valencia, Spain

In the EU and UK, novel foods are foods that were not used for human consumption to a significant degree within the Union and the UK before 15 May 1997 [1,2]. In the EU, it is regulated by Regulation (EU) 2015/2283 [1]. In Australia and New Zealand, novel foods are non-traditional foods that are also regulated by the Food Standards Code [3]. This field, novel foods and novel food ingredients, is also covered by the U.S. Food and Drug Administration (FDA) [4], as ensuring foods are safe for the population is essential. Generally, a novel food could be a traditionally, locally consumed food or an ethnic food in a country that is a new food to others. Additionally, new kinds of food and substances used in food are being developed based on common and new proteins, oils, extracts, fibers, starch, etc., deriving from algae, fungi, insects, plants, by-products, etc. New food development will drive progress in promoting innovative healthy foods and could accommodate new lifestyles and increased consumer awareness [5,6]. The perception of sustainability could also have a positive impact on the acceptance of novel foods [7].

In this context, this Special Issue was launched, focused on potential novel sources of disease prevention that fit into the concept of novel food. This is aligned with the idea that health, nutrition, sustainability, safety, and food security are crucial pillars to reach some of the targets proposed by the UN’s sustainable development goals of “Good health and well-being”, “Zero hunger”, and “Responsible consumption and production”. The link between these topics and novel foods and bioactive compounds would be evident if such foods and compounds really improve health and nutrition, as demonstrated by research, while addressing global food security and sustainability challenges.

The contributions are depicted below and include the following focuses: plant extracts and agroindustrial byproducts (e.g., coffee pulp) with bioactive properties, non-traditional cereals and derived products, which can contribute to the recommended dietary intake of particular nutrients or serve as sources of bioactive peptides, the use of insect powder for food applications, and underutilized but edible tropical fruits with interesting phytochemical profiles. Further research, industrial deployment, and safety assessments will contribute to the success of investigated sources of novel foods. Ultimately, the novel food should be proven to produce the claimed effect, be safe, and be profitable for the producer. It seems that the dietary supplement and functional food markets are increasing [5], so this could be a driver for innovation in this field. In addition, market studies to determine consumer demand will be crucial. For example, Günden et al. suggested that it is crucial to assess the extent to which consumers adopt these food innovations and develop new eating habits, as there are barriers such as novel food/novel food technology neophobia (i.e., hesitation or avoidance regarding the consumption of this type of food) [6]. According to Laureati et al. [7], appropriate product design and well-managed communication about the sustainability benefits and health claims related to such foods could be beneficial for consumer acceptance.
